# Early-life risk factors which govern pro-allergic immunity

**DOI:** 10.1007/s00281-024-01020-x

**Published:** 2024-07-27

**Authors:** Catherine Ptaschinski, Bernhard F. Gibbs

**Affiliations:** 1 Department of Pathology, University of Michigan, Ann Arbor, USA; 2 Mary H. Weiser Food Allergy Center, University of Michigan, Ann Arbor, USA; 3 School of Psychology and Life Sciences, Canterbury Christ Church University, North Holmes Road, Canterbury, Kent, CT1 1QU UK

**Keywords:** Allergy, Perinatal, Anaphylaxis, Food allergy, Microbiome

## Abstract

Allergic diseases affect up to 40% of the global population with a substantial rise in food allergies, in particular, over the past decades. For the majority of individuals with allergy fundamental programming of a pro-allergic immune system largely occurs in early childhood where it is crucially governed by prenatal genetic and environmental factors, including their interactions. These factors include several genetic aberrations, such as filaggrin loss-of-function mutations, early exposure to respiratory syncytial virus, and various chemicals such as plasticizers, as well as the influence of the gut microbiome and numerous lifestyle circumstances. The effects of such a wide range of factors on allergic responses to an array of potential allergens is complex and the severity of these responses in a clinical setting are subsequently not easy to predict at the present time. However, some parameters which condition a pro-allergic immune response, including severe anaphylaxis, are becoming clearer. This review summarises what we currently know, and don’t know, about the factors which influence developing pro-allergic immunity particularly during the early-life perinatal period.

## Introduction

The prevalence of allergy has consistently risen in the past century, ranging between 10 and 40% of the world population according to the World Allergy Organization (WAO), with 40–50% sensitization rates amongst school children to one or more common allergens [1]. Since the mid-1990s food allergies have risen sharply and hospitalizations for severe allergic reactions (anaphylaxis) in England and Wales rose by over 600% between 1992 and 2012 [2]. Food allergic hospitalizations are highest in infants, underlining the critical conditioning of pro-allergic immunity in early childhood, including the perinatal period [2]. This is typified by recent findings demonstrating that tolerance to peanuts and subsequent peanut allergy risk reduction significantly takes place at 4–11 months compared to introducing peanuts at 5 years of age [3, 4]. The immune system in early childhood obviously plays a pivotal role in the subsequent development of allergic disease.

Several genetic aberrations, such as filaggrin loss-of-function mutations, are associated with allergy and it has long been known that the risks of developing allergies are partially hereditary, with a 30–50% chance of a child developing allergies with one allergic parent, which increases to 60–80% with two allergic parents. But the example given above clearly shows that this genetic predisposition can be either mitigated or compounded depending on exposure to certain microorganisms, nutrients, chemicals, drugs and life-style factors. This review summarises what we currently know about the factors which influence developing immunity leading to allergy, particularly during the early-life perinatal period. However, while many genetic and environmental determinants have been identified which moderate allergies, we know relatively little about the details of early human adaptive immune responses. Here, recent findings from animal models of allergy have shed light on some of the unknowns and these too are included in this review, in the hope that it will stimulate the unmet need for a better understanding of human perinatal pro-allergic immunity.

## Development of the perinatal immune system

Perinatal immunity undergoes striking changes because of differing fundamental requirements associated with each stage of development. Whereas the foetal immune system needs to tolerate maternal alloantigens and avoid inflammatory pathways which could hinder the normal development of vital tissues and organs, the sudden exposure to environmental pathogens after birth requires robust innate and adaptive immunity to cope thereafter (reviewed in [5]).

Foetal immunity is largely mediated by the innate immune system, including neutrophils, monocytes, macrophages and dendritic cells, though with diminished functional properties, alongside reduced capacity for complement activation. Neutrophils, for example, display reduced migration through the endothelium and bactericidal functions, and phagocytic ability is also impaired until shortly after birth [6, 7]. Foetal and neonatal immune cells, such as plasmacytoid dendritic cells, display reduced levels of pro-inflammatory cytokines such as interferon (IFN)-α and -β, IL-12 and TNF-α, while producing more anti-inflammatory cytokines including IL-10 and TGF-β [8, 9]. Although diminished innate and inflammatory immune responses potentially render the foetus susceptible to infections, they crucially help to avoid spontaneous abortions and damage to the developing lungs and other tissues [10].

In terms of early adaptive immunity, both CD4 + and CD8 + positive T cells are present in the thymus from the second trimester onwards, but their subsequent priming and activation is generally diminished. This may be due to reduced number and function of antigen presenting cells. For example, it was shown that cord blood myeloid-type dendritic cells (mDC) are reduced in number compared to adults and express less HLA class II as well as CD80 and CD86 on the cell surface [11]. Nonetheless, when activated, foetal CD4 + T cells are primarily skewed towards a regulatory (Foxp3 + CD25 +) T cell (Treg) phenotype upon stimulation with alloantigens due to the effects of TGF-β, thereby crucially contributing to self-tolerance [12].

Although the foetal immune system is capable of eliciting weak Th1 immune responses, its later development into the neonatal phase is highly skewed to Th2 immunity, especially upon foreign antigen activation [13]. This Th2-biased response continues after birth, and is explained in part by hypermethylation of the promotor region of *IFNG* gene in T cells, preventing transcription and skewing to Th1, whereas Th2-promoting regions are hypomethylated and poised for activation [14, 15]. The balance between early tolerogenic (Treg-mediated) immunity *versus* Th2 immunity has obvious repercussions with respect to the development of allergic diseases and other Th2-mediated pathologies.

Humoral adaptive immunity is also strikingly undeveloped, especially in utero and neonatal stages. B cell expressions of CD80/86 and CD40 in infants aged under 2 months are reduced and are therefore less able to respond to activation by helper T cells in the presence of an antigen [16]. They also lack the capacity for affinity maturation of antibodies due to limited somatic hypermutation [17]. At birth, a large proportion of B cells are of the B1 type, which secrete IgM specific to a limited range of antigens (and of low-affinity), whereas B2 cells predominate several months after birth and are capable of producing a more diverse repertoire of immunoglobulins such as IgM, IgA as well as IgE. Interestingly, IgE can also be detected in cord blood, especially in association with allergic disease. However, the source of IgE could be maternal, since the immunoglobulin can cross the placental barrier when complexed with IgG [18]. Whether this maternal IgE can then activate mast cells is currently an area of debate [19].

Mast cells and basophils are the primary drivers of allergic reactions as they have the high-affinity receptor for IgE. Mouse studies have shown that mast cells appear in the skin during embryonic stages, but do not appear in other tissues until after birth [20, 21]. However, the skin mast cells that are present appear to be immature and unable to respond to IgE [19]. Conversely, results from both human and mouse have shown that basophils are present in circulation from birth [22, 23]. Furthermore, these basophils block the function of dendritic cells in newborn mice, thereby preventing the development of a Th1 immune response [23]. Taken together, the neonatal immune system is primed to respond to Th2 stimuli.

## Genetic factors

Family history is a strong risk factor in the development of atopy and allergy, indicating a clear role for genetics in allergic disease. There are numerous genetic variations that have been associated with higher risk of allergy. Several of these variations are due to mutations in barrier proteins, especially in the skin. As the skin is often the first site of allergic inflammation, these have been widely studied. The most well-characterized is filaggrin (*FLG*). Mutations in this protein are associated not only with predisposition to atopic dermatitis, but also with allergic asthma, allergic rhinitis, and food allergy [24]. Mice with *FLG* mutations have also been commonly used to understand the role of this protein in allergic disease [25]. However, *FLG* mutations do not contribute to asthma development without the presence of atopic dermatitis, indicating a relationship between these two pathologies. This relationship is discussed below.

In addition to barrier genes, several mutations in genes that code for key immune processes are also implicated in allergic disease. For example, genome-wide association studies (GWAS) studies have identified several susceptibility genes in asthma, allergic rhinitis, and atopic dermatitis. These include key Th2-associated cytokines such as IL-33 and TSLP, as well as the IL-33 receptor [26, 27]. These cytokines are important in driving the Th2 response in allergic disease. However, there are also genetic variations within the effector Th2 cells themselves. For example, single nucleotide polymorphisms in the *IL13* gene have been found to be associated with increased risk of food allergy and greater levels of circulating IgE [28]. STAT6 is a transcription factor that drives IL-4-mediated responses, and variations in this gene have been linked to nut allergy [29]. Additionally, a variant in the IL-4 receptor associated with asthma has been shown to convert regulatory T cells into Th17 cells, thereby enhancing disease [30]. Other Treg variations have been found in cord blood, including *IL10* polymorphisms [31]. It should be noted that individually, most gene variants have a low odds ratio when assessing allergy risk, however it is the interplay between genetics and environmental factors that is likely to drive disease.

## Maternal factors affecting allergic disease

Allergic disease has long been thought to be due to allergen exposure after birth, however several recent studies have indicated that susceptibility can be traced to factors that occur in utero. While maternal antibody transfer has already been mentioned, several other maternal factors including diet, stress, and other environmental exposures have been shown to significantly increase allergy risk.

### Stress

Maternal stress has recently been implicated in a number of childhood outcomes, although much of this has been linked to childhood behaviour [32]. The link between maternal stress and allergic disease has long been suspected by using a number of murine models of allergic disease [33]. This models have used both acute and chronic stress protocols, including direct delivery of corticosterone in drinking water [34] and have consistently found that maternal stress results in increased Th2 responses and airway hyperreactivity in offspring [35]. However, this has been more difficult to assess in humans, although several groups have investigated maternal stress and immune development. More recently, a systemic review and meta-analyses has shown an increased correlation between maternal stress and allergic disease [36]. This includes the finding that anxiety and depression during pregnancy resulted in increased risk of atopic dermatitis, allergic rhinitis, wheezing, and asthma, as well as increased circulating IgE levels, and that the risk was greatest when the maternal stress occurred during the third trimester [36]. The mechanism behind this is currently unclear but is likely due to the hypothalamic–pituitary–adrenal axis, whereby stress hormones such as cortisol and adrenaline are released into the abdomen, allowing for transmission to the foetus [37].

### Diet

Maternal diet is another area of research that has undergone renewed interest in recent years when considering the development of allergic disease. While maternal folate has long been understood in developmental processes, more recent work has also considered the role of this vitamin in immune development. While folate is necessary in the first trimester to prevent neural tube defects, current evidence suggests that excess folate in the third trimester may increase the risk of allergic disease. Much of this is due to its role as a methyl donor, which is needed for proper DNA and histone methylation to occur. Differential DNA methylation in both CD4 + T cells and antigen presenting cells from cord blood is altered in high- *vs* low-folate diets during the third trimester [38]. Neonatal CD4 + T cells from cord blood of infants born to women with very high folate levels also exhibit increased histone acetylation on *GATA3* promotor regions. As histone acetylation is a known activating epigenetic mechanism, this may indicate a pre-disposition to Th2 responses [39]. A meta-analysis looking at folate levels found that the risk of respiratory allergic diseases was increased in pregnant women who took folate supplements [40].

Conversely, prenatal exposure to various nutrients via maternal diet may protect from allergic disease during childhood, although a number of analyses on this front have been inconclusive. Most promising in vitamin D supplementation, where reduced risk of wheeze was seen in children before three years of age [41], while other dietary factors such as long-chain fatty acids seem to have limited effectiveness when taken prenatally [41]. Epidemiological data suggest that vitamin D exposure can be protective against allergic disease. These data are primarily from studies looking at allergy severity, especially food allergy. Studies have found more severe anaphylaxis in children further from the equator, where vitamin D levels are lower, as well as in children born in winter, compared to spring or summer [42]. More direct evidence comes from a study demonstrating higher confirmed oral-food challenge in infants with lower vitamin D levels in both peanut and egg allergy [43]. Recently, a 15-year follow-up found maternal supplementation resulted in decreased asthma incidence in children up to 6 years old [44]. In this context, high vitamin D may promote regulatory T cell development and IL-10, offering protection from allergy [45].

Conflicting data exist on other maternal dietary interventions, such as fruit and vegetable intake, and the development of childhood allergies, although a diet rich in probiotics such as yogurt consumption is correlated with lower allergic risk [46]. Interestingly, maternal consumption of peanut, milk, and wheat during pregnancy is associated with decreased risk of allergies and asthma [47]. This has also been observed in animal models of peanut allergy, where low-dose peanut exposure during pregnancy and lactation of female mice provided protection against peanut allergy in offspring [48]. This protection is mediated in part by enhanced DNA methylation at the *IL4* promotor, thereby inhibiting Th2 responses [49].

### Environmental exposures

The association of prenatal environmental chemical exposure and adverse outcomes following birth have been studied in several contexts, including allergic disease. Bisphenol A, used in the manufacture of plastics, has been associated with an increased risk of asthma, especially when exposure occurs during the third trimester [50]. Addition exposure to perfluorochemicals, used in polymer coatings associated with water- and stain-resistant materials, has been linked with increased IgE and atopic dermatitis [51]. Although polychlorinated biphenyls have been banned in many places, these chemical compounds persist in the environment and prenatal exposure has been linked with asthma and atopic dermatitis [52]. Exposure to heavy metals such as nickel, chromium, lead, copper, mercury, and arsenic are also implicated in atopy development, and risks from exposure to these metals continue after birth (reviewed in [53]). One of the most important prenatal environmental exposures is to cigarette smoke. Numerous studies in humans and animal models have demonstrated a link between respiratory issues such as asthma following maternal smoking and have been comprehensively reviewed elsewhere [54, 55].

Commonly used medications used during pregnancy have also been associated with increased allergy risk. Paracetamol, a commonly used analgesic and fever-reducer, has been linked to childhood asthma in longitudinal cohort studies [56]. During paracetamol metabolism, the antioxidant glutathione is depleted, and insufficient glutathione may drive T cells to a pro-allergy Th2 phenotype [57]. Additionally, in utero aspirin exposure, while often recommended for a number of pregnancy-related conditions such as pre-eclampsia, is also associated with increased asthma risk [58].

## Postnatal exposures linked to allergic disease

The postnatal period is a time of rapid immune development as interaction with microbes and other environmental factors drive maturation. While many of the factors that contribute to the development of allergic disease prenatally carry into early life, the first month to years of childhood present a separate set of challenges for the immune system. These exposures can either increase the risk of allergic disease, or can provide protection, often through the development of tolerance.

### The hygiene hypothesis

The hygiene hypothesis was developed in the late 1980s in an attempt to explain the exponential increase in allergic diseases in the second half of the twentieth century [59]. This hypothesis suggests that a clean environment that lacks exposure to microbes early in childhood increases the risk of allergic disease by modulating the maturation of the immune systems away from a Th2 bias. While this hypothesis originally identified infections as a driver of allergic disease, more recent work has focused instead on the role of commensal bacteria in the development of the immune system. This has been supported by population-based observations. Initial studies focused on farm populations, where it was noted that children raised among farm animals (particularly cattle) had lower levels of atopy and allergy [60]. This has been further refined to show that direct contact with farm animals in early life is protective against allergic disease, even among populations with similar genetic backgrounds [61]. Further studies demonstrated that having pets is also protective; those studies went on to analyse microbes in the dust of houses and found a greater diversity in those houses with indoor/outdoor pets [62]. Indeed, the development of the gut microbiome and its associated metabolites is now thought to play an important role in the development of the immune system and protection from allergic disease [63]. This hypothesis is further supported by observations that vaginal delivery results in a lower risk of allergy compared to caesarean section, as the initial bacteria that colonize the newborn infant are derived from either the vaginal tract or the skin [64]. Infants born vaginally often have a more diverse microbiota, which is important in the development of balanced and/or tolerogenic responses early in life [65]. This revised focus of the hygiene hypothesis may offer a chance for early-life intervention in atopic individuals.

### Microbiome

As discussed above, the focus of the hygiene hypothesis has recently been on the development of the microbiota early in life. Data from children monitored for several years after birth suggest that the gut microbiota composition at one month old predicts future atopy and asthma later in life [63]. Several studies have looked not only at the microbiome composition, but also the metabolites produced by commensal microbes in response to diet and the development of the immune response. These studies have focused on *Clostridiales* species and dietary fibre intake and have found that fibre consumption in the presence of *Clostridiales* results in the production of short-chain fatty acids [66].

Thorburn et al. demonstrated that short-chain fatty acids such as acetate, which can cross the placenta, promote T regs and subsequently prevents the development of allergic airways disease in mice [67]. This underlines the notion that maternal diet plays a substantial role in the risk of developing asthma alongside epigenetic input. Arpaia et al. observed similar Treg-promoting effects were also observed with the short-chain fatty acids butyrate and propionate [68]. Polyunsaturated fatty acids (PUFAs), such as omega − 3 fatty acids which are commonly found in fish oils, may also have protective effects since they decrease dendritic cell function by modulating NF-κB activity [69]. In contrast, PUFAs such as omega-6, common in vegetable oils, have been shown to be associated with increased risk of asthma [70]. However, this association was observed to be sex-specific since male children were at highest risk of developing asthma (or wheeze) if maternal asthma was present alongside a high plasma ratio of omega-6 *vs.* omega-3 PUFA [70].

Other studies have shown that *Lactobacillus* species promote a diverse microbiota that preferentially produces favourable metabolites, such as short-chain fatty acids, and decrease Th2-associated cytokine production [71]. Short-chain fatty acids also regulate the immune response through epigenetic programming through changes in histone acetylation and DNA methylation [72, 73]. These processes also promote Treg function and help to maintain local hyporesponsiveness.

The importance of the microbiota in allergic disease is also highlighted by several observations regarding the use of antibiotics, especially early in life when the microbiome is still developing. Antibiotic use is common in the first three years of life, and studies in humans and mice are beginning to uncover the ways in which antibiotics during this time can perturb development [74]. In a neonatal mouse model, antibiotic exposure resulted in a decrease in intestinal beta diversity [75]. A longitudinal study in children up to three years of age found that antibiotic use resulted not only in less diversity, but also in an increase in antibiotic resistant genes [76]. A very large retrospective study of nearly 800,000 showed that early-life antibiotic use correlated with the development of a variety of allergic conditions, and that exposure to more than one class of antibiotics increased this risk [77]. Moreover, even maternal antibiotic exposure in pregnancy was demonstrated to be significantly associated with respiratory allergic diseases in early childhood [78]. While antibiotic use is often critical and lifesaving, these studies may serve to make more informed decisions about antibiotic class and duration in the future.

### The dual allergen hypothesis

The link between atopic dermatitis and food allergy has long been recognized, and is termed the dual-allergen hypothesis. This states that sensitization to food allergens may occur through skin exposure, as opposed to oral exposure [79]. This is supported by the observation that many children react to food on first known oral exposure [80]. As atopic dermatitis is a Th2-mediated disease that results in production of IL-33 and TSLP, which are known to drive IgE-mediated food allergy, the idea that allergen sensitization could occur through the skin has received a great deal of attention [81]. Murine studies have suggested a role for IL-33 in the cross-talk between the skin and the intestine in this route of sensitization [82].

Atopic dermatitis affects up to 12 percent of infants, and is often associated with mutations in skin barrier genes [83]. In those infants, the incidence of food allergy is up to six times higher than infants without eczema [84]. However, these skin barrier mutations do not increase the risk of food allergy independently of atopic dermatitis, indicating a role for environmental factors in this association. Recently, detergent exposure has been an area of active research regarding epithelial barrier function [85]. While detergents alone are unlikely to contribute to allergy development, studies have shown that detergents such as SDS can enhance sensitivities over a threshold level to detect disease [86]. Previous experiments in mice have also found that the presence of SDS can enhance immune responses in the lymph node, possibly though increased dendritic cell trafficking [87]. Detergents can also impact skin barrier function, so even in the absence of mutations in barrier genes, atopic dermatitis can develop [88]. Similar disruptions in lung and intestinal barrier function have been found following detergent exposure [89, 90].

### Early life exposure to allergens

As mentioned above, maternal ingestion of foods such as peanut may provide a degree of protection from allergy in infants. These recent findings are in contrast to previous recommendations that suggested avoidance of allergens during pregnancy and breastfeeding. Similarly, older guidelines recommended avoidance of peanuts in children thought to be high-risk for the development of peanut allergy. However, ongoing studies pointing to the importance of developing early-life tolerance resulted in many rethinking this advice [91]. The most well-known study to date has been the LEAP study (Learning Early about Peanut Allergy). This study found that infants at high risk for developing peanut allergy, such as those with eczema or with egg allergy, were significantly less likely to develop peanut allergy when peanut was introduced early into the diet [92]. This study demonstrated that oral tolerance to food antigens could be induced by early exposure. Furthermore, a follow-up study (LEAP-On), found that this tolerance was sustained even after peanut consumption was stopped [93].

Since the publication of the LEAP study, a number of other clinical trials using other foods have been conducted with some promising, though mixed, results. Several of these studies have used early introduction of egg, as this is a common food allergy early in life. While most studies did show a decrease in the incidence of egg allergy, most were not statistically significant (reviewed in [94]). One study that did demonstrate a significant protection focused on those infants with eczema and combined egg introduction with eczema treatment. However, other groups found that many infants were already sensitized to egg at four to six months of age, indicating that early egg introduction may not be viable for several groups [95]. A larger trial, Enquiring About Tolerance (EAT trial), aimed to introduce the most common food allergens at three months of age alongside breastfeeding. These allergens included peanut, egg, cow’s milk, sesame, whitefish, and wheat. While this study did find that early introduction was safe in a general population, it did not find significant differences in the development of food allergies [94]. However, a follow-up study that focus solely on high-risk infants, such as those with a food sensitization or eczema, did find a lower level of allergy to one or more foods [96]. The results of the EAT follow-up study, along with the clinical trial for egg introduction in high-risk infants with eczema, suggest that early introduction of allergens may be beneficial in those infants that are likely to develop food allergies, but that the effect in the general population may not be as pronounced.

### Viral infections

Several respiratory viral infections have been associated with asthma exacerbations, however there is also a potential role for early-life viral infections in the development of allergic lung disease. The most well-studied of these is respiratory syncytial virus (RSV). This viral infection is the leading cause of bronchiolitis in children, and infects nearly all infants by two years of age [97]. Several studies have found that severe RSV infection requiring hospitalization increases the risk of developing asthma by three- to five-fold [98]. Early in life, RSV infection results in a Th2 or Th17 skewed response, as opposed to the Th1 response often elicited by viral infection. This is driven primarily by release of the alarmins, especially TSLP, from the airway epithelium, which activate a Th2 response through CD4 + T cells and type 2 innate lymphoid cells [99]. Clinical studies have shown that children with severe disease have a dampened Th1 response compared to those with mild disease, and that this immune signature persists following recovery from infection [100]. Follow-up studies in animal models have demonstrated that RSV infection of neonatal mice results in long-term TSLP expression and enhanced Th2 responses to allergen models even several weeks after infection [101]. While there is a debate as to whether the association between RSV and asthma is causative or due to similar underlying Th2 biases in the immune system, the long-term immunological changes seen after RSV infection suggest that there may be a causative role in the development of wheeze and asthma. Furthermore, mouse models have found that early-life infection with RSV results in changes in the microbiome, resulting in less diversity in both the lung and gut microbiota following infection [102]. A systemic review of the literature also found difference in both the gut and lung microbiome in RSV-infected patients compared to healthy controls [103]. While these results are difficult to analyse as samples from infected patients were not available prior to acquiring RSV, they do suggest that further longitudinal studies such as birth cohorts may benefit from collected microbiome samples upon RSV infection of infants.

## Concluding remarks

**Fig. 1:**
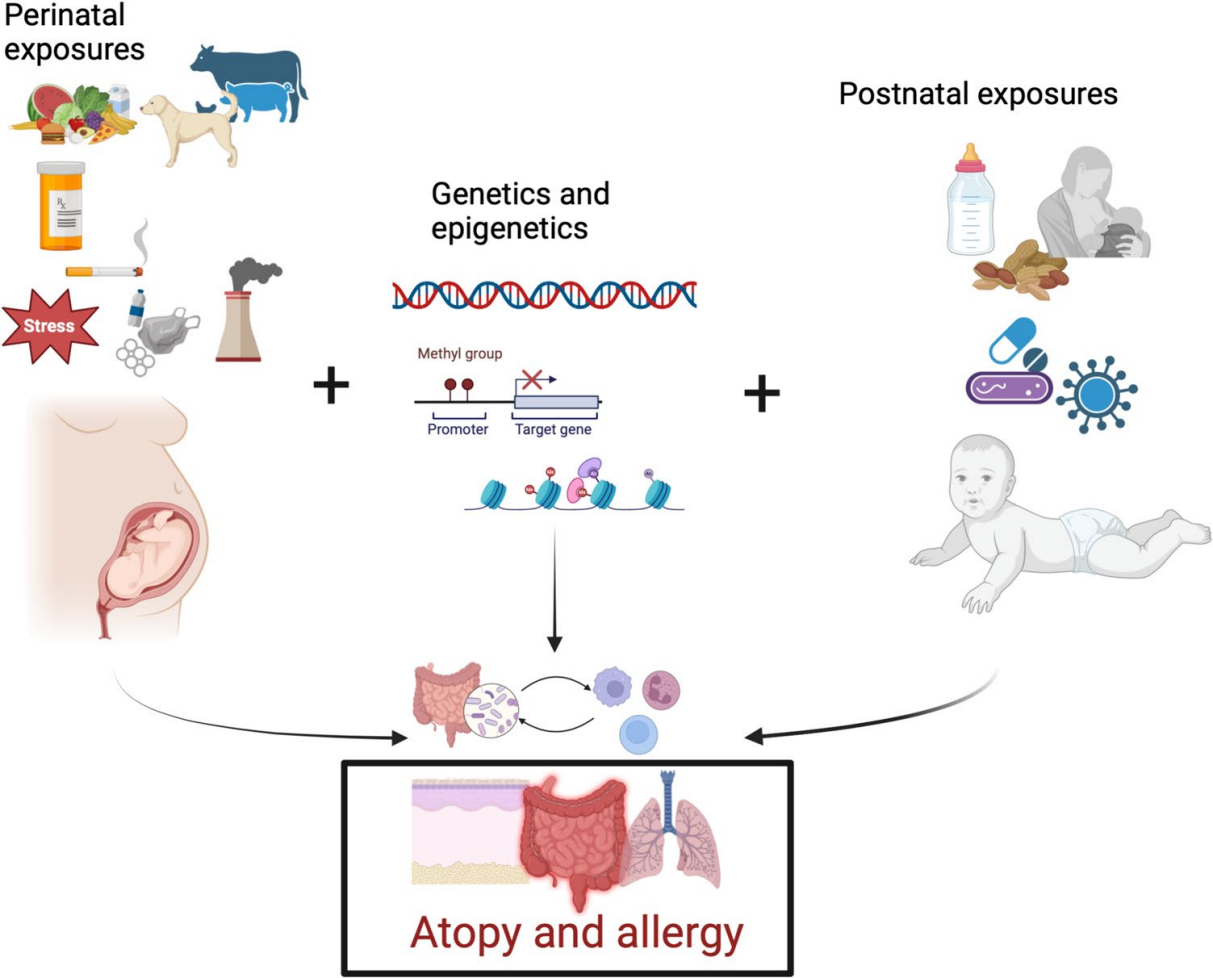
The development of allergic diseases is influenced by the interplay of environmental factors and genetics. Prenatal exposure to pets and farm animals, as well as maternal nutrient intake, may offer protection from allergy. Conversely, maternal stress and exposure to pharmaceuticals, cigarette smoke, and pollutants may contribute to disease. These exposures interact with genetic and epigenetic factors, which in turn impact immune maturation in response to environmental pressures after birth, including mode of feeding, infections and antibiotic use, and allergen exposure. Together, these interactions impact the establishment of the microbiome and the developing immune system. These complex interactions early in life are important risk factors in the development of allergic diseases. Created with BioRender.com

Allergic diseases have diverse phenotypic manifestation but are all connected by an underlying Th2-driven immune response. A number of genetic factors underly the ability of the immune response to switch toward an anti-allergic Th1 phenotype, although genetics alone do not account for the increase in allergy over the last 50 to 70 years. Instead, the interaction of environmental factors prior to birth and in early life with underlying genetic susceptibility drives this complex family of pathologies. The immune system is Th2-skewed at birth and early in life, increasing the risk for developing atopy and allergy. Understanding the multiple factors that contribute to disease may lead to the implementation of preventative strategies, which are preferable to treatment after disease has been established. These factors are summarised in Fig. 1. This approach has already been met with some success in early peanut introduction in high-risk children. While this approach has not been universally successful, the collective increase in knowledge of early-life risk factors is likely to provide more positive results in the future.

## Data Availability

Data sharing is not applicable to this article as no datasets were generated or analysed during the current study.
